# Travelling through the Natural Hierarchies of Type I Collagen with X-rays: From Tendons of Cattle, Horses, Sheep and Pigs

**DOI:** 10.3390/ma16134753

**Published:** 2023-06-30

**Authors:** Alberta Terzi, Nunzia Gallo, Teresa Sibillano, Davide Altamura, Annalia Masi, Rocco Lassandro, Alessandro Sannino, Luca Salvatore, Oliver Bunk, Cinzia Giannini, Liberato De Caro

**Affiliations:** 1 Institute of Crystallography, National Research Council, 70125 Bari, Italy; alberta.terzi@ic.cnr.it (A.T.); teresa.sibillano@ic.cnr.it (T.S.); davide.altamura@ic.cnr.it (D.A.); roberto.lassandro@ic.cnr.it (R.L.); cinzia.giannini@ic.cnr.it (C.G.); 2 Department of Engineering for Innovation, University of Salento, 73100 Lecce, Italy; annalia.masi@unisalento.it (A.M.); alessandro.sannino@unisalento.it (A.S.); l.salvatore@typeone.it (L.S.); 3 Typeone Biomaterials Srl, Via Europa 167, 73021 Calimera, Italy; 4 Paul Scherrer Institute, 5232 Villigen PSI, Switzerland; oliver.bunk@psi.ch

**Keywords:** biomaterial, type I collagen, tissue regeneration, WAXS, SAXS, mechanical properties, tendon, thermal analysis

## Abstract

Type I collagen physiological scaffold for tissue regeneration is considered one of the widely used biomaterials for tissue engineering and medical applications. It is hierarchically organized: five laterally staggered molecules are packed within fibrils, arranged into fascicles and bundles. The structural organization is correlated to the direction and intensity of the forces which can be loaded onto the tissue. For a tissue-specific regeneration, the required macro- and microstructure of a suitable biomaterial has been largely investigated. Conversely, the function of multiscale structural integrity has been much less explored but is crucial for scaffold design and application. In this work, collagen was extracted from different animal sources with protocols that alter its structure. Collagen of tendon shreds excised from cattle, horse, sheep and pig was structurally investigated by wide- and small-angle X-ray scattering techniques, at both molecular and supramolecular scales, and thermo-mechanically with thermal and load-bearing tests. Tendons were selected because of their resistance to chemical degradation and mechanical stresses. The multiscale structural integrity of tendons’ collagen was studied in relation to the animal source, anatomic location and source for collagen extraction.

## 1. Introduction

Regenerative medicine is a medical field that attempts to re-establish both structure and function of damaged tissues and organs. One of the tools used to trigger the regeneration consists of the fabrication of biomimetic, biocompatible and biodegradable scaffolds, that are then implanted into the site of injury, stimulating the regeneration. The challenge of tissue engineering (TE) is the production of a material with a topographical and geometric shape that mimics as much as possible the native tissue, a necessary condition for the regeneration to obtain a new tissue with the desired shape and function. While the extracellular matrix (ECM) is a complex assembly of molecules, scaffolds currently used in regenerative medicine are only a minimum matrix with few components that, however, must be similar to the native multilevel structure of the matrix [1]. Additionally, if the original hierarchical structure is difficult to achieve artificially, the assembly of macromolecules in a synthetic scaffold sheds light on the fundamental role of the multiscale architecture and topography on the final regeneration process. One of the widely used biomaterials for scaffold fabrication is type I collagen, a fibrous protein common in the human body that confers mechanical support, strength and toughness to the tissue, and directs cellular phenotype thanks to its biochemical and topographical triggers [2]. It holds a specific hierarchically organized structure. Starting from the molecular scale, a type I collagen molecule is composed by three coiled α-helices (two α1 chains and one α2 chain) forming a right-handed triple helix with a rod-like shape (diameter of about 1.1 nm, length of 300 nm) [3]. The helices are coiled with a staggering of one residue, one with respect to the other. Helicoidal portions of collagen molecules are side by side with non-helicoidal parts that are placed at both C and N termini and comprise 16–26 amino acidic residues [4]. The molecular stabilization is guaranteed by hydrogen bond formation within the molecular backbone and between polar amino acid residues and water molecules that surround the triple helix forming a hydration cylinder [5]. Two different models were proposed to describe the helical pattern of the molecular structure of collagen. Both of them have been assessed by X-ray investigations. The Rich and Crick model [6] represents a 10/3 triple-stranded helical model with 10 triplets in 3 turns and a single inter-chain hydrogen bond per triplet (10/1 helical symmetry). In each triplet 3.33 residues/turn, the helical rise of 28.9 Å and helical pitch of 86 Å are assumed. In 2006, Okuyama et al. [7] proposed an alternative model based on X-ray studies of a synthetic crystal. The model is a 7/2 triple-helical structure with seven triplets in 2 turns, 3.5 residues/turn, an axial repetition of 20 Å and a pitch of 60 Å. Both models are now accepted, in particular the 10/3, which is typical for the structure of collagen being poor in proline, while the 7/2 is proline-rich [7,8]. From a supramolecular point of view, five collagen triple helices are laterally staggered forming the fibril in a three-dimensional liquid crystalline lattice in which molecules have a quasi-hexagonal geometry and are twisted in a right-handed arrangement. The molecular lateral packing distance is about 1.6–1.8 nm, related to the hydration of the tissue. The quarter-staggered unit of five collagen molecules laterally packed with a 1.5 nm distance leads to the formation of two regions of different electron density distribution: a high electron density region due to the overlap of five packed collagen molecules (about σ D wide) called σ wide region (σ < 1, σ ~ 0.46) and a low electron density distribution part due to the overlap of only four molecules and one gap (about (1 − σ) D wide). The σ region possesses a packing density 20% higher than (1 − σ) [9]. The repetition of these alternate zones along the fibrillary axis leads to the characteristic fibrillary periodicity of about D ~ 67 nm (0.46 D ~ 30.82 nm (overlap) + 36.18 (gap), for σ = 0.46). The periodic electron density distributions are stabilized by the formation of a charge–charge interaction of the positive amino acid residues of the lateral chains [10]. With the static AFM nanoindentation, Minary-Jolandan et al. [11] observed that the gap (1 − σ) and the overlap (σ) regions possess a wide gap in both elastic and energy-dispersion properties. In particular, an average elastic modulus of about ∼2.2 GPa in the overlap region (σ) and ∼1.2 GPa for the gap region (1 − σ) was observed. Thus, the mechanical heterogeneity along the fibrillary axis is directly related to these two regions that form the whole D period. The physiologic or pathologic alteration of their ratio, thus of their width, can vary not only the electron density distribution but also the mechanical elasticity and stiffness of the collagen itself and the tissue in which it lays.

The wide-angle X-ray scattering (WAXS) technique has demonstrated itself to be suitable for the investigation at the atomic scale of type I collagen in biomaterials and native tissues, allowing the inspection of the fundamental features of molecules and their spatial structure. The small-angle X-ray scattering (SAXS) technique is effectively employed for the evaluation of supramolecular characteristics of collagen at the nanoscale, namely, fibrillary orientation and periodicity value and gap–overlap ratio, correlating these with the functional properties of the fibrils [8,12,13,14]. X-ray diffuse scattering may shed light on the dynamic disorder, typical of liquid crystals, associated with the spatial distribution in the tissue [15].

Thanks to the type I collagen abundance in tissues, it is extensively extracted from several animal sources, such as bovine, porcine, piscine and equine. Tendons are gaining more appeal for medical industries as they are collagen-rich tissues (about 60–85% of the dry mass), with low cellularity (less than 5% of the whole tendon volume) and blood vessels and nerves. Tendons are part of the musculoskeletal system and connect muscles to bones permitting the transmission of the muscle’s mechanical load generated for the movement of the body [16]. Their crucial function allows joint stability and low energy dispersion during the movement. 

While tendons are primarily made of type I collagen, there are also non-collagenic proteins and glycans, such as elastin, proteoglycans (PGs) and glycosaminoglycans (GAGs), able to retain a high number of water molecules. This part of the tissue is called “ground matter” and helps the sliding of collagen molecules and fibrils during traction and tension [17]. Tendons possess specific shapes and sizes in relation to their function, for instance, extensor tendons are round shaped while flexors are flat [8]. Collagen fibrils in tendons are mainly oriented along the direction of forces application and are crimped to confer flexibility to the tissue; they can also reach 150 nm in diameter in humans [18]. The fibrils oriented along the longer axis of the tissue are called T-type and are those which may absorb a major part of forces. Thin fibrils of around 10 nm diameter are called C-type and are orthogonally oriented and embedded between T-type fibrils. C-type fibrils keep the tissue in the anatomic position during movement. It is well known that the diameter of fascicles vary in relation to the tendon location, while the number of fascicles can vary in relation to the exercise [19,20].

With the aim to find the best tendon source for the extraction of type I collagen suitable as a biomaterial for fabrication of medical devices in relation to the final tissue to regenerate, we analyzed, at both molecular and fibrillar scales, type I collagen extracted from bovine, porcine, ovine and equine tendons. Measurements were performed by wide- and small-angle X-ray scattering. Furthermore, we have compared the collagen structure, spatial organization and arrangement of all the samples with the denaturation and degradation temperature analyses (differential scanning calorimetry, DSC) and load-bearing tests of the tissues. Finally, we investigated the differences between flexor and extensor tendons from equine sources, evaluating also the possibility to select a tendon not only in relation to the animal source but also to the anatomic localization. The study gives an indication about the animal source to use for collagen extraction, in relation to the overall properties to be achieved in the final biomaterial for tissue regeneration applications.

## 2. Materials and Methods

### 2.1. Materials 

Deep extensor tendons from equine (age: 12–18 months), bovine (age: 18–24 months), porcine (age: 8–9 months) and ovine (age: 2–4 months) of unknown sex (Figure S1 (Supplementary Material)) were gently provided from a local slaughterhouse, rinsed three times with distilled water and stored at −20 °C in polyethylene bags. Prior to use, samples were thawed at 4 °C. Distilled water was obtained from Millipore Milli–U10 water purification facility from Merck KGaA (Darmstadt, Germany).

### 2.2. Wide- and Small-Angle X-ray Measurements

Wide- and small-angle X-ray scattering analyses (WAXS and SAXS) were carried out at the X-ray Micro Imaging Laboratory (XMI-L@B) of the Institute of the Crystallography of the CNR [21]. The instrument is composed by a Fr-E+ SuperBright copper anode MicroSource (λ = 0.154 nm, 2475 W) coupled by a focusing multilayer optics Confocal MaxFlux (CMF 15–105) to a three-pinhole camera. The beam size at the sample was about 0.2 × 0.2 mm^2^ during both WAXS and SAXS data collection. Shreds of tendons from bovine, porcine, ovine and equine of about 1 cm × 1 cm, were kept in Ultralene^®^ sachets sealed (SPEX™ SamplePrep 3525, 65 Liberty street, Metuchen, NJ, USA). In such a way, the tissues remain wet tissues during measurements. We used Ultralene^®^ because it is a thin film that allows to obtain a uniform transmission of X-rays. As it has a good chemical and heat strength, it is widely employed in X-ray analysis of wet samples. The samples prepared as described, were then set on the sample holder and located in a chamber in a vacuum (0.1 mbar) for the signal collection. WAXS data were recorded by an Image Plate (IP) detector (250 × 160 mm^2^, 100 µm effective pixel size) mounted 10 cm far from the sample, collecting data in a range of 1.8 ÷ 21 Å in the direct space (from 0.3 to 3.5 Å^−1^ in the reciprocal space). SAXS data were acquired in a range of 3 ÷ 105 nm in the direct space (from 0.006 to 0.2 Å^−1^ in the reciprocal space), mounting the IP detector ~2 m far from the samples. The X-ray data were extracted by the offline RAXIA reader and data were elaborated by SAXSGUI 2.05 and SUNBIM 3.0 software [22]. Detector–sample distances were calibrated by using the powder of the Si NIST standard sample for WAXS and the Ag Behenate standard sample for SAXS, both placed in Ultralene^®^ sachets.

Four samples are indicated in Table 1; for each sample, three WAXS and SAXS signals from three points were acquired. Both WAXS and SAXS patterns were acquired at each point for 900 s. Further SAXS analyses of the extensor equine tendon were performed at cSAXS beamline of the Swiss Light Source in Villigen, Switzerland [23]. This set-up is composed of a liquid N2 cooled fixed-exit Si (111) monochromator with a bendable second crystal for horizontal focusing, a bendable mirror for the rejection of higher X-ray energies and vertical focusing, a sample holder on a motorized 2D translation stage, a 7 m long evacuated flight tube between the sample and the detector for SAXS measurement and a Pilatus 2M detector downstream of the scattered X-ray beam for data collection [24]. SAXS data were collected in a range of 3.4 ÷ 314 nm in the direct space (from 0.002 to 0.18 Å^−1^ in the reciprocal space) and measurements were performed using a monochromatic X-ray beam (λ = 0.09537 nm, E = 14.6 keV) focused down to about 10 μm (vertical) and 30 μm (horizontal) in size. Samples were studied in Ultralene sachets sterilized in ethanol 70% (*v*/*v*) and washed in phosphate-buffered saline (PBS) to remove ethanol. The data collection was performed in continuous lines with 50 μm distance between lines. SAXS data were collected with the detector positioned 7098 mm downstream the sample. The acquired SAXS data were analyzed using multi-modal [23] and segmentation approaches [25] to find the most representative profiles for each investigated sample. According to the characteristic extraction of the inflection points of the scattering curves of SAXS/WAXS measurements, i.e., Bragg peaks and slope variations, the methods classify scattering curves. The statistical approach allows us to find similarities between signals and to classify them into a few representative clusters, with no need for models or prior sample knowledge, that can be used for further analysis. Experimental data were calibrated based on standard measurements of silver behenate and folded into 1D profiles after azimuthal integration.

### 2.3. Mechanical Behavior

Tendon constitutive bond strength was evaluated using a Zwick-iLine universal testing machine (Zwick/Roell, Ulm, Germany). Samples of 5 × 20 mm were cut along a tendon’s long axis, incubated in 0.01 M PBS at room temperature for 1 h and tensile tested until reaching a failure A preload of 0.1 N and a load speed of 0.1 mm/s. A loading cell of 1 kN was applied [26]. The Young modulus (E), the break stress (σ_max_) and the break strain (ε_r_) were measured and were estimated as reported elsewhere [13,26,27,28]. Six different samples were tested for each tendon type.

### 2.4. Thermal Properties

DSC measurements were performed with a Q2000 Series DSC from TA Instruments (New Castle, DE, USA). Tendon samples of about 5–7 mg were sealed into hermetical aluminum pans and subjected to a heating ramp from 5 °C to 200 °C, in inert nitrogen atmosphere (50 mL min^−1^) [26]. Scan speed was set at 5 °C/min. The reference was performed with an empty pan. The endothermic phenomena peak temperatures were measured as the mid-point of the endothermic transition [28,29]. The energy required for the endothermic phenomena was calculated as the area under the peak [28,29]. The OriginPro software (Origin–Lab version 8, Northampton, MA, USA) was chosen for DSC data analysis. Each tendon type was analyzed in triplicate.

### 2.5. Statistical Analysis

Data obtained from both mechanical and thermal tests were expressed as mean ± standard deviation. Statistical significance of data was determined using the one-way analysis of variance (ANOVA) test and two-tailed t Student test. Differences were considered significant for *p* values less than 0.05.

## 3. Results

### 3.1. Ultrastructural Analyses (WAXS and SAXS)

**Figure 1:**
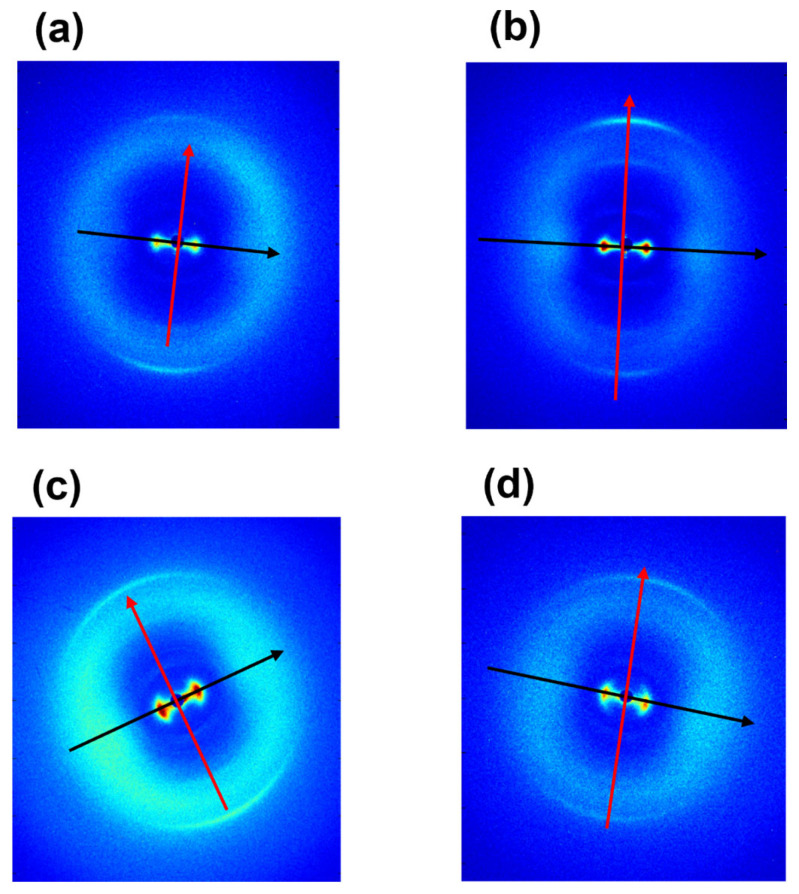
Two-dimensional WAXS diffraction patterns of equine extensor (**a**), bovine (**b**), ovine (**c**) and porcine (**d**) tendons. The equatorial and meridional directions are marked with black and red arrow, respectively.

**Figure 2:**
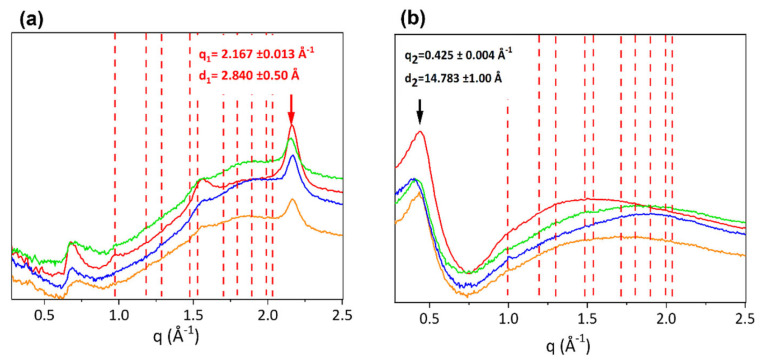
One-dimensional diffraction profiles integrated along the meridional (**a**) and equatorial (**b**) directions. Profiles of equine extensor (blue), bovine (red), ovine (green) and porcine (orange) tendons are overlapped and both the meridional (**a**) and equatorial (**b**) peaks are marked with red and black arrows, respectively. Red dotted vertical lines represent Ultralene^®^ characteristic peaks.

Figure 1 shows 2D WAXS patterns of samples of equine extensor (a), bovine (b), ovine (c) and porcine (d) tendons. The anisotropic diffraction patterns reveal two typical orthogonal directions, specifically the equator (black) and the meridian (red). After radial integration, the 2D WAXS data were converted into the meridional (Figure 2a) and equatorial (Figure 2b) 1D profiles. 

The peak along the meridional direction (Figure 2a) at q_1_ = 2.167 ± 0.013 Å^−1^, corresponding to d_1_ = 2.84 ± 0.50 Å, is the distance among two close amino acid residues along the axis of the triple helix (axial turn). The major peak along the equatorial direction (Figure 2b), identified at q_2_ = 0.425 ± 0.004 Å^−1^ corresponding to a d distance of about d_2_ = 14.78 ± 0.150 Å, refers to the lateral packing distance between collagen molecules.

Besides the crystalline component of collagen structure, the amorphous part of the fibrous protein is also crucial, meaning by amorphous that all the molecular segments dispersed in a non-crystalline ordered phase. Indeed, both crystalline and amorphous component orientations influence the mechanical properties of fibers. As shown in Figure 2 in 2D patterns of ovine (c) and porcine (d) tendons, between the equatorial and the meridional signals, a broader circular signal is detected in comparison to equine extensor (a) and bovine (b), indicative of a major amorphous phase in ovine and porcine tendons. This is a region characterized by high structural heterogeneity and water amount, that acts as a plasticizer and appears to be related to the distance between the collagen in the tendon and the skeleton [30]. 

For the investigation of the order at the molecular scale, the extent of crystalline domains was studied by measuring the FWHM of the Gaussian fitting of the equatorial peak for each sample, related to the molecular lateral packing within the tissue (Table 2). In particular, the lower the FWHM value, the wider the extent of crystalline domains, thus the molecular order within the material. As shown in Table 2, bovine and porcine tendons have a smaller FWHM than equine and ovine, thus a wider crystalline domain.

**Figure 3:**
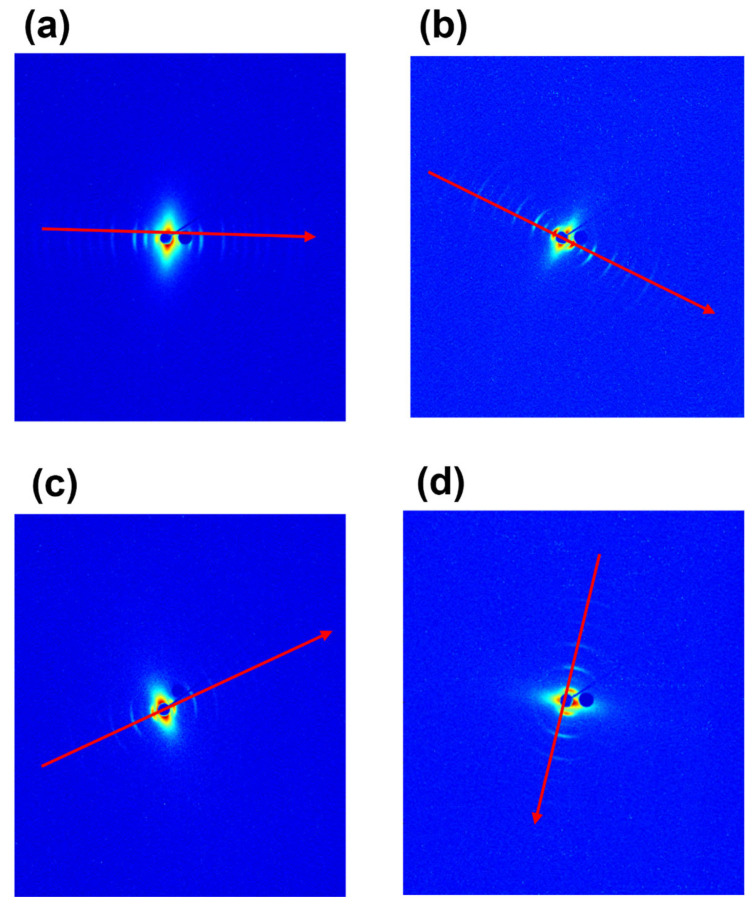
Two-dimensional SAXS diffraction patterns of equine extensor (**a**), bovine (**b**), ovine (**c**) and porcine (**d**) tendons. The meridional direction is marked by a red arrow.

The supramolecular fibrillary structure of type I collagen in tendons was analyzed by SAXS at the XMILab. Data are shown in Figure 3 in which the 2D diffraction anisotropic patterns display the typical arcs of an oriented fiber (the meridian direction, along the fiber axis, is marked by a red arrow). 

**Figure 4:**
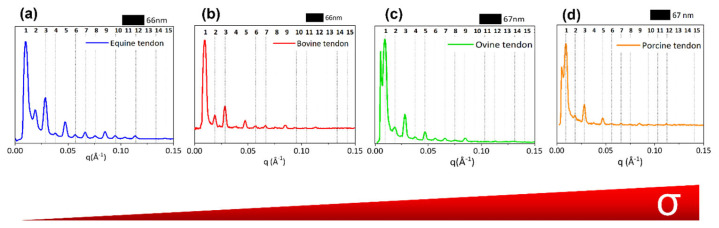
The 1D diffraction profiles integrated along the meridional direction are shown for equine extensor (**a**), bovine (**b**), ovine (**c**) and porcine (**d**) tendons. Profiles are sorted following the increasing σ value (increased overlap size).

After folding the 2D SAXS data into 1D profiles (Figure 4), equally spaced peaks are identified (marked by vertical bars). The axial periodicity extracted from these peaks corresponds to the characteristic fibrillary periodicity of about 67 nm. The difference in the expected D value of about 67 nm can be due to both the hydration state of the tissue and the specific animal species. In fact, small differences of electron density distribution values were observed across all samples, that is, bovine 66 nm, equine extensor 66 nm, ovine 67 nm and porcine 67 nm tendons. Furthermore, collagen in the equine (Figure 4a), bovine (Figure 4b) and ovine (Figure 4c) tendons have visible peaks up to high orders, so the periodicity is maintained across multiple orders.

The 1D profiles have been analyzed also in terms of relative intensity of the diffraction orders, directly related to the electron density along the fibrillary axis. A systematic dumping of the first two even peaks (2nd and 4th order) is observed in all samples, particularly greater in bovine (b), ovine (c) and porcine (d) tendons. Noteworthy, the dumping effect disappears at a wider angle, that is, at higher orders. Going into detail, in our previous work [9], we mentioned that the axial period can be divided into the two parts of characteristic electron density: the overlap (σ) about 0.46 D wide and the gap − σ) of about 0.54 D. These values may undergo slight variations due to slight changes of the collagen triple helix staggering, as a function of the different mechanical function/load of tendons in different animal species. The increase of σ value up to about 0.5 D corresponds to the increase of the extent of the overlap region (five staggered molecules) and to a reduction of the gap (four staggered molecules), with respect to the expected values of overlap (σ = 0.46 D) and gap (1 − σ = 0.54 D). We observed that, when σ increases and (1 − σ) decreases, both reaching values close to 0.5 D, this leads, in combination with the electron density projected on the fiber axis fulfilling certain symmetry operations [9], to substantial systematic absences in the collagen diffraction pattern. By a kinematical approximation, the damping of the even interference peaks in the axial (meridional) direction when σ and (1 − σ) are both 0.5 D is expected, particularly on the 2nd (black line), 4th (blue line) and 6th order (magenta line), as shown in Figure S2 (Supplementary Material). Thus, as shown in Figure 4, the dumping effect on the 2nd and 4th peaks appears to be greater in pig (d) tendon than in equine (b) or bovine (b) tendons. We also assume that the dumping is not visible at wider angles as higher scattering vectors allow us to appreciate also small differences between the axial extension of the overlapping and gap regions, with respect to the exact value of a half period or the symmetry of the electron density profile [9], and consequently, the dumping effect of even orders higher than the fourth disappears.

**Figure 5:**
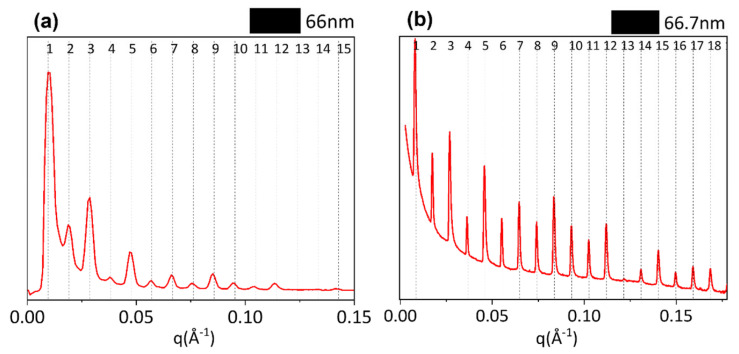
Comparison between 1D SAXS profiles obtained from equine extensor tendon at the XMI Lab (**a**) and at Swiss synchrotron SLS (**b**).

In order to analyze nanostructural features as close as possible to the hydrated natural conditions and to compare structural information with mechanical behavior analyzed in a wet environment, further analyses were conducted at the SLS that allowed us to perform X-ray measurements also at ambient pressure. From the comparison between XMILab (a) and SLS (b) data collected on the same equine extensor tendon, a similar trend of the relative intensity was observed for both measurements, although the SLS pattern shows more diffraction orders, with a greater visibility, as expected due to higher incident X-ray flux and higher resolving power. However, a slight decrease in the periodicity value in XMILab (66 nm) than in SLS (66.7 nm) was measured together with a major dumping effect on the 2nd and 4th diffraction peaks in Figure 5a. This could be a little effect due to dehydration of the material when collecting data in a vacuum at the XMILab. 

### 3.2. Mechanical Properties

**Figure 6:**
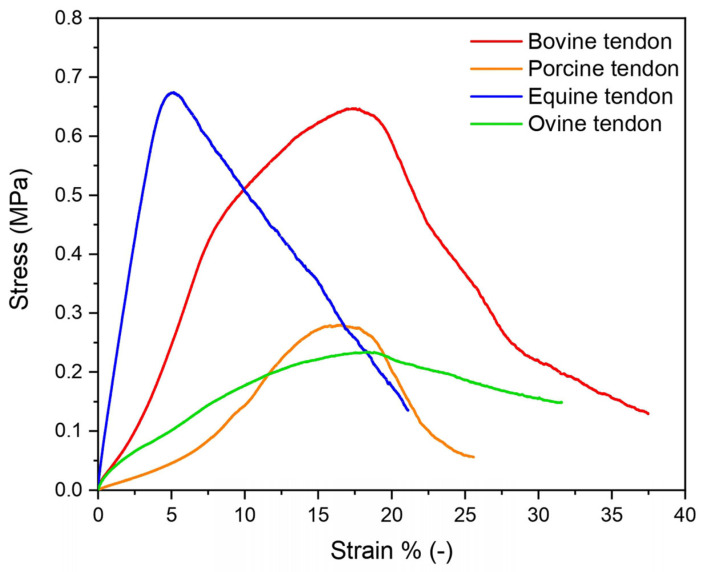
Representative stress–strain curves of equine (blue), bovine (red), porcine (orange) and ovine (green) tendons.

Tendons’ mechanical response depends on their highly organized hierarchical structure that reflects their micro- and nano-organization. To investigate tendons’ structural properties on the macroscopic level, the mechanical response of all of them was evaluated by means of a tensile test. Figure 6 shows the stress–strain curves of equine (blue), bovine (red), porcine (orange) and ovine (green) tendons. The equine extensor tendons were revealed to have the highest E modulus, followed by bovine, ovine and porcine tendons (Table 3), reflecting the different natural load-bearing capacities. As known, mechanical behavior reflects the motor habits of these animals. Thus, equine tendons were much stiffer than those of other animals as they must support the horse’s leg movements during fast running, in addition to the fact that they have to support the weight of a large-size animal. Similarly, tendons of cattle, which move less than horses but are still large-size animals, had a high modulus. Next were tendons of ovine, which are much smaller in size than horses and bovines and therefore need more support at a lower load. Finally, pigs are characterized by the lowest modulus. Similarly, the maximum stress registered was similar for equine and bovine and was found to be three times higher than that of porcine and ovine tendons. Very different values were registered compared to those found in literature, due to the different preconditioning and strain rates applied in this study from other literature [31]. Tendons had a viscoelastic behavior for which a strong test parameter dependence exists: an increase in the stress and strain at failure was registered with a strain rate increase [32,33]. Moreover, it should be mentioned that tendon properties are strongly dependent not only on animal type but also on age, sex, site, feeding, living condition and general health status. 

### 3.3. Thermal Properties

**Figure 7:**
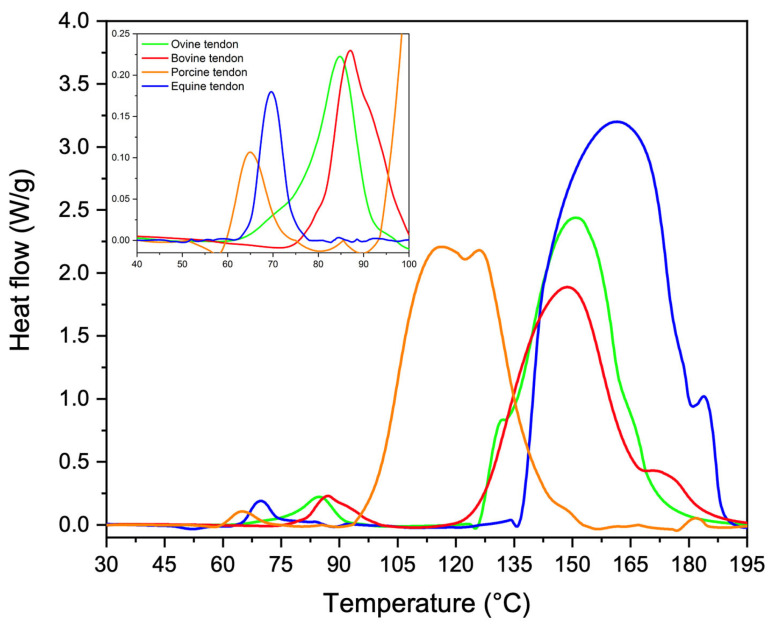
Representative thermograms of equine extensor (blue), bovine (red), porcine (orange) and ovine (green) tendons.

Molecular-level differences were revealed by DSC. As reported in Figure 7, from thermograms of equine extensor (blue), bovine (red), porcine (orange) and ovine (green) tendons, two phenomena were revealed: a minor endothermic event at about 60–100 °C and a greater endothermic event at about 100–180 °C. In Table 4, a registered phenomenon mean peak temperature and enthalpy was reported. The first phenomenon could be attributed to the packing structure of collagen molecules (i.e., denaturation) [34,35], that was found to have almost the same peak temperature for all analyzed tendons (*p* > 0.05). Despite that, a statistically significant difference was found in the required enthalpy which was higher for bovine tendons, followed by the ovine and equine extensors, and lastly by the porcine tendons (*p* = 0.006). The second phenomenon could be assigned mainly to the breaking of the direct hydrogen bonds between alpha chains [34] and it was found to be at about 110–170 °C, with no statistically significant differences. Additionally, in this case, calculated enthalpies required for the transition were found to be significantly different (*p* = 0.0003). In particular, the endothermic phenomenon of the equine extensor tendon was the one that required the highest energy, which was found to be about 1.5-fold higher than the one of the porcine tendon, about 1.8-fold higher than the one of the ovine tendon and about 2.1 higher than the one of the bovine tendon. Thus, although denaturation and degradation temperatures of tendons were quite the same, the different amount of energy required suggested the presence of different types or number of inter-chain or intra-chain bonds. According to these evidence, equine extensor tendons should be characterized by a higher number of inter-chain or intra-chain bonds, followed by porcine, ovine and bovine.

## 4. Discussion

Tendons are connective tissues with low cellularity and a great content of type I collagen (about 60–85% of the dry mass). The yield of protein after the extraction processes for the fabrication of biomaterials appears to be higher from tendon sources than from other tissues [36]. The aim of this paper was the investigation of structural characteristics of type I collagen in tendons from different animal sources for future applications in tissue engineering. From ultrastructural analyses, an anisotropic diffraction signal of the protein at both atomic and nanoscales in all samples was observed which was a marker of molecular and fibrillary preferred orientation along the forces application direction (orientational anisotropy). Particularly, WAXS was employed for the investigation of collagen molecular structure and arrangement. Data show both crystalline and amorphous components of the fiber at molecular scale. The crystalline component represents specific structural features of the triple helix, such as residue distance along the helical axis of the molecule, which was found equal to about d = 2.9 ± 0.02 Å, typical of the 10/3 model of collagen molecule (2.9 Å), also known as Rich and Crick model [6], which describes a 10-fold helical symmetry in which 3.33 triplets are repeated per turn and the axial repeat is about 28.6 Å. Further information about the regular and periodic distribution of triple helices in the space is contained in the crystalline structure and can be analyzed, for instance, by investigating the FWHM of the equatorial peak representing the lateral packing distance of collagen helices. For the samples analyzed here, the lateral distance of triple helices (d = 14.78 ± 0.15 Å) is similar, that is, it is not related to the animal source. However, the degree of crystallinity and thus the degree of regular and periodic distribution of triple helices in the tissue is different. For its evaluation, the analysis of the FWHM of the equatorial peak (molecular lateral packing) in each sample was performed.

Based on our data analyses, we conclude that molecules are arranged in wider crystalline domains in bovine, porcine and equine extensor samples than in ovine tendons. Interestingly, the amorphous component exhibiting a partially random distribution of molecules in a space enables tendons to retain water, thereby helping to stabilize the crystalline component. In fact, it confers viscoelastic properties due to the dynamic formation of water bridges in which water molecules continuously exchange with others in the network around collagen structures [37]. In large-size animals such as horses or cattle, the tissue is characterized by wide crystalline domains dispersed within little amorphous regions that give it stability and reduce the brittleness of the tendon, helping the sliding of collagen structures at all the length scales during traction and tension. In these samples also, the highest E-modulus are observed, reflecting the natural load-bearing capacity of large-size animals. Interestingly, bovine and equine tendons, although structurally similar, require a different amount of energy for collagen degradation. In particular, energy for collagen degradation is higher in equine tendons than in the other samples including cattle, highlighting a different number of inter- and intra-chain bonds that affect physical properties.

Looking at the supramolecular structure of type I collagen (SAXS measurements), in equine extensor and bovine tendons, the periodicity of the electron density distribution is better defined than in the other samples, probably due to the necessity to sustain and transmit mechanical forces that act along the length of the tissue, thus in relation to the height/weight of the animal. In support of this hypothesis, we observe that in pig tendons, shorter than bovine and equine tendons, the nanometric arrangement of collagen fibrils appears to be less organized. In particular, pig fibrils appear characterized by a wider overlap region (σ > 0.46 D) with respect to the other samples; indeed, the dumping effect of the 2nd and 4th diffraction peaks is more visible than in the other animals. The decrease of the gap–overlap ratio is related to a more rigid structure which can reduce the strength of the tissue. Indeed, a wider gap region (1 − σ; ε ∼ 1.2 GPa) than the overlap (σ; ε ∼ 2.2 GPa) guarantees a higher elasticity and therefore the strength of the tissue. In fact, from mechanical tests, it was observed that the maximum stress that pig tendons can support is 3 times less than in equine and bovine and the denaturation enthalpy is lower than in the other animals, leading to a tissue of less strength. For these reasons, we assume that, since a long tendon is mainly subjected to uniaxial stress compared to the multidirectional stress to which a shorter tendon and a heavy animal, such as a pig, can be subjected, the nanofibrillar organization is crucial to maintain structural integrity when the tissue length is large, which means that, in this case, it may be influenced more by the height of the animal than by its weight and by the stress type. Furthermore, the relationship between the gap and overlap zones into fibrils, and therefore the distribution of electronic density, appears to significantly influence the mechanical characteristics, as a wider gap region with less overlap seems to guarantee good strength properties to the tissue. From the cross-analyses of nanoscale fibrils at SLS and XMILab, we were able to determine if specific features which we observed in samples are intrinsic of the tissue or just affected by its hydration state. In fact, water molecules content can affect collagen structure and stabilization. By comparing completely (Figure 5b) and partially wet (Figure 5a) samples, we observed that even a tiny decrease in water content, which means less intra- and inter-fibrils water-mediated H-bonds (water bridges), induces a slightly smaller periodicity of the electron density distribution and a major dumping effect on the 2nd and 4th diffraction peaks, thus a more rigid structure due to the increase of overlap region size (σ > 0.46 D) with respect to the fully hydrated tissue. 

In summary, equine extensor tendons are characterized by a significantly higher number of inter-chain or intra-chain bonds and elastic modulus than determined for the other samples. Porcine and ovine tendons can take less stress than equine and bovine ones. This correlates to a higher molecular overlap along the fibrillary axis, as especially noted for porcine tendons. Thus, even though the building blocks are the same, the hierarchical structure differs, and tendons are structurally optimized for their function, which includes the load-bearing patterns of long animals with more directional forces than for short ones, and for the weight and thus load on the tendons. Keeping this structural information in mind, this work can be suitable to choose not only the animal source to be used for collagen extraction but also the anatomic location of the tendon, in relation to the overall properties to be achieved in the final biomaterial for tissue regeneration. As an outlook, we aim at a systematic comparison of tendons and potentially other biomaterials across different farm animals and locations in the animal to provide a better understanding for the selection of biomaterials from widely available farm animals. Moreover, as decellularized tissues are widely used as grafts for tissue regeneration, alternative to scaffolds in regenerative medicine, we propose our approach for these biomaterials. In particular, decellularization protocols may negatively affect the structure and mechanical behavior of the extracellular matrix, also altering the cell responses and load-bearing features. In order to explore this relationship, it would be necessary to study the decellularization protocols and the stability of the different collagen raw sources, to improve protocols and obtain a final tissue as similar as possible to the original one.

## Figures and Tables

**Table 1:** Samples analyzed.

Samples
Bovine tendon
Porcine tendon
Extensor equine tendon
Ovine tendon

**Table 2:** WAXS results on tendon samples.

	Equatorial Direction			Meridional Direction	
Tendon	Lateral q_2_-Spacing (Å^−1^)	Lateral d_2_-Spacing (Å)	FWHM (Å^−1^)	Axial Turn q_1_-Spacing (Å^−1^)	Axial Turn d_1_-Spacing (Å)
Extensor equine	0.397 ± 0.001	15.826 ± 0.040	0.124 ± 0.003	2.169 ± 0.001	2.896 ± 0.001
Bovine	0.429 ± 0.001	14.646 ± 0.034	0.114 ± 0.004	2.162 ± 0.001	2.906 ± 0.001
Ovine	0.415 ± 0.001	15.140 ± 0.040	0.134 ± 0.002	2.156 ± 0.001	2.914 ± 0.001
Porcine	0.426 ± 0.001	14.749 ± 0.034	0.111 ± 0.002	2.168 ± 0.001	2.898 ± 0.001

**Table 3:** Mechanical properties of tendons. Results are expressed as mean value ± SD.

Tendon	E (MPa)	σ _max_ (MPa)	ε_r_ % (−)
Equine extensor	13.8 ± 3.0	0.6 ± 0.2	4.9 ± 1.5
Bovine	4.8 ± 1.2	0.7 ± 0.1	20.3 ± 4.7
Ovine	2.7 ± 1.5	0.3 ± 0.1	17.1 ± 3.2
Porcine	0.9 ± 0.2	0.3 ± 0.1	14.8 ± 3.1

**Table 4:** Thermal properties of tendons. Results are expressed as mean value ± SD.

Tendon	T_I_ (°C)	ΔH_I_ (J/g)	T_I_ (°C)	ΔH_I_ (J/g)
Equine extensor	70 ± 10	16 ± 4	171 ± 19	1422 ± 203
Bovine	86 ± 9	33 ± 7	151 ± 18	673 ± 84
Ovine	84 ± 8	23 ± 8	154 ± 22	789 ± 92
Porcine	65 ± 12	9 ± 3	112 ± 17	912 ± 63

## Data Availability

Not applicable.

## Supplementary Materials

The following supporting information can be downloaded at: https://www.mdpi.com/article/10.3390/ma16134753/s1, Figure S1. Schematic description of anatomic part of the extensor tendon explored here. Figure S2. Systematic absences in the collagen diffraction pattern. In a kinematical approximation, for the electron density function described in [9], the n-th diffraction order is missing if n times sigma is exactly equal to an integer number.
