# Supplementary material for: Travelling through the Natural Hierarchies of Type I Collagen with X-rays: From Tendons of Cattle, Horses, Sheep and Pigs
Source: Materials (Basel). 2023 Jun 30;16(13):4753. doi: 10.3390/ma16134753 (PMC10342676; doi:10.3390/ma16134753)
Supplement: Supplementary file 1 [file materials-16-04753-s001.zip › materials-2445222-supplementary.pdf]

*Supplementary Material*

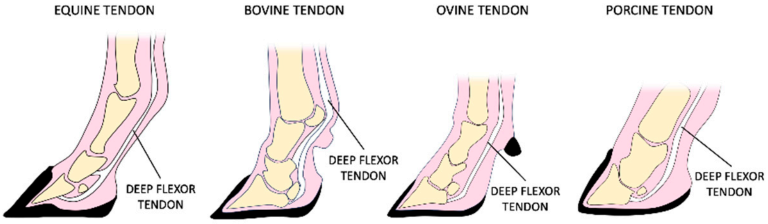


**Figure S1.** Schematic description of anatomic part of the extensor tendon explored here.

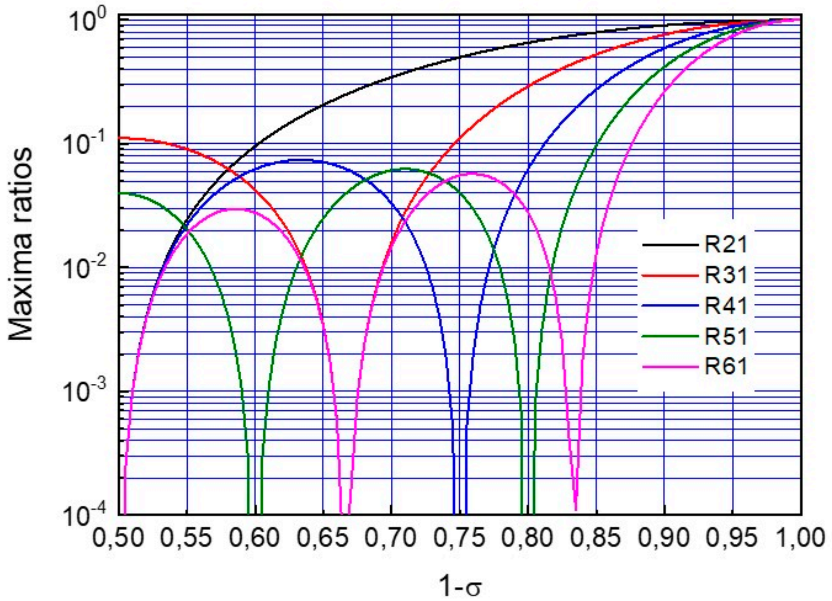


**Figure S2.** Systematic absences in the collagen diffraction pattern. In a kinematical approximation, for the electron density function described in [1], the n-th diffraction order is missing if n times sigma is exactly equal to an integer number.

[1] Giannini, C.; De Caro, L.; Terzi, A.; Fusaro, L.; Altamura, D.; Diaz, A.; Lassandro, R.; Boccafoschi, F.; Bunk, O. Decellularized Pericardium Tissues at Increasing Glucose, Galactose and Ribose Concentrations and at Different Time Points Studied Using Scanning X-ray Microscopy. IUCrJ 2021, 8, 621–632, doi:10.1107/S2052252521005054.
